# Astaxanthin from Shrimp Cephalothorax Stimulates the Immune Response by Enhancing IFN-γ, IL-10, and IL-2 Secretion in Splenocytes of *Helicobacter Pylori*-Infected Mice

**DOI:** 10.3390/md17070382

**Published:** 2019-06-26

**Authors:** Sergio Davinelli, Heidi Mikkelsen Melvang, Leif Percival Andersen, Giovanni Scapagnini, Michael Engelbrecht Nielsen

**Affiliations:** 1 Department of Medicine and Health Sciences “V. Tiberio”, University of Molise, Via de Sanctis s.n.c, 86100 Campobasso, Italy; 2 Department of Clinical Microbiology, Copenhagen University Hospital (Rigshospitalet), Blegdamsvej 9, 2100 Copenhagen, Denmark; 3 Department of Research and Development, FB Dermatology Ltd., Borupvang 5C, 2750 Ballerup, Denmark

**Keywords:** astaxanthin, *Helicobacter pylori*, infection, inflammation, cytokines

## Abstract

Infection with *Helicobacter pylori* is a critical cause of gastrointestinal diseases. A crucial host response associated with *H. pylori* infection includes gastric inflammation, which is characterized by a sustained recruitment of T-helper (Th) cells to the site of infection and distinct patterns of cytokine production. Adequate nutritional status, especially frequent consumption of dietary antioxidants, appears to protect against infection with *H. pylori*. The aim of the present study was to investigate whether astaxanthin (AXT) from shrimp cephalothorax may modulate cytokine release of splenocytes in *H. pylori*-infected mice (*n* = 60). Six- to eight-week-old female mice were divided into three groups (*n* = 20 per group) to receive a daily oral dose of 10 or 40 mg of AXT for six weeks. After six weeks, a trend toward interferon gamma (IFN-γ) upregulation was found (40 mg; *p* < 0.05) and a significant dose-dependent increase of interleukin 2 (IL-2) and IL-10 (both *p* < 0.05) was observed. These results suggest that AXT induces higher levels of IL-2 and a shift to a balanced Th1/Th2 response by increasing IFN-γ and augmenting IL-10. We concluded that AXT may influence the pattern of cytokines during *H. pylori* infection.

## 1. Introduction

*Helicobacter pylori* is a gram-negative bacterium that infects more than half of the world’s population [1]. *H. pylori* colonization usually occurs in childhood and persists throughout life, causing chronic gastritis, peptic ulceration, gastric adenocarcinoma, and primary gastric lymphoma [2]. Long-term gastric inflammation is the hallmark of chronic *H. pylori* infection and is thought to underlie several gastrointestinal diseases. The severity of gastric inflammation varies, depending on the host immune response against this bacterium. However, colonization by *H. pylori* recruits host immune system cells such as macrophages, neutrophils, and lymphocytes to the gastric mucosa [3,4]. Although the inflammation of the gastric mucosa depends mainly on T-helper 1 (Th1) cell responses, it has been acknowledged that *H. pylori* infection also induces the differentiation of anti-inflammatory T-helper 2 (Th2) cells [5,6]. Th1 and Th2 subsets exhibit distinct patterns of cytokine secretion and the Th1 phenotype is characterized by a predominance of interferon gamma (IFN-γ), which contributes to the persistence of inflammation and to the inhibition of a possibly beneficial Th2 response. In particular, increased levels of IFN-γ establish a Th1-dominant microenvironment, inhibiting interleukin 2 (IL-2) activity, which plays an essential role in the priming of Th2 responses and protection against the infection [7,8]. However, there is evidence that a shift from a Th1-polarized state to a mixed Th1/Th2 response, characterized by IL-10 production, may enhance the clearance of the *H. pylori* [9,10]. Dietary antioxidants have gained attention due to their immunomodulatory properties, particularly to slow down Th1-type activation pathways and elicit a combined secretion of both Th1 and Th2 cytokines [11,12]. Moreover, observational studies have reported a correlation between a low intake of dietary antioxidants and an increased risk of *H. pylori* acquisition [13,14]. Astaxanthin (AXT) is a xanthophyll carotenoid that possesses antioxidant, anti-inflammatory, and immune-enhancing properties. This compound is ubiquitous in nature, primarily biosynthesized by microalgae/phytoplankton and found in the marine environment as a red-orange pigment common to many crustaceans such shrimp, crabs, and lobster [15]. Several experimental studies have suggested that AXT from the microalga *Haematococcus pluvialis* may reduce bacterial load associated with *H. pylori* infection as well as inhibit colonization and inflammation. Additionally, it was observed that AXT extracted from *H. pluvialis* induced a shift from a Th1-dominant state to a balanced Th1/Th2 response [16,17]. Shrimp shells are an excellent source of AXT, particularly concentrated in the cephalothorax and when extracted using fish oil [18]. However, there is a lack of data on the immunomodulatory effects of AXT obtained from this source during *H. pylori* infection. In this study, we investigated the influence of different oral doses of AXT from shrimp cephalothorax on the cytokine release of splenocytes in *H. pylori*-infected mice. 

## 2. Results

### 2.1. Astaxanthin Hydrolysis

**Figure 1:**
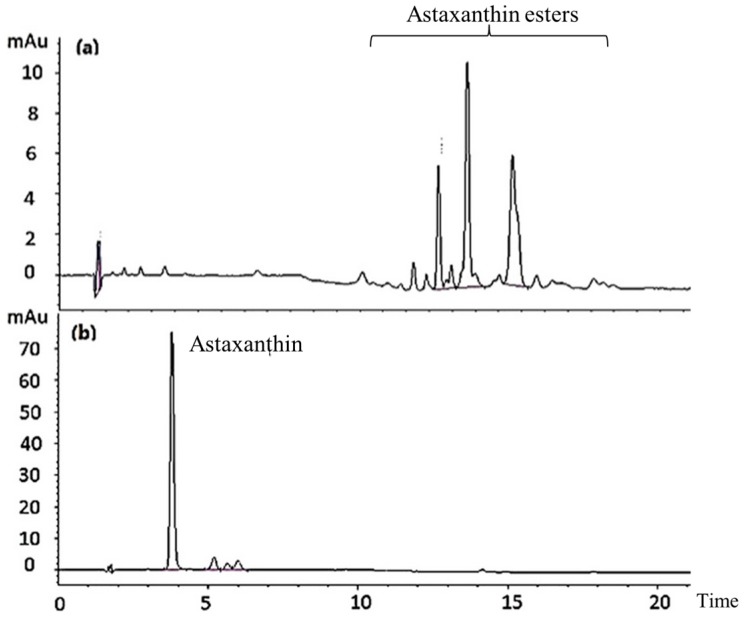
HPLC chromatograms of AXT in shrimp cephalothorax before (**a**) and after (**b**) hydrolysis treatment.

The saponification of AXT esters is a well-known hydrolysis treatment method after AXT extraction. Figure 1 shows the separation of fractions. Free AXT was identified according to the retention time of an AXT standard. Data from the high-performance liquid chromatography (HPLC) assay suggested that fractions with retention time between 10 min and 20 min disappeared after hydrolysis. The AXT fraction, with retention time at 4 min, increased remarkably after hydrolysis in comparison with those before hydrolysis. These results indicate that saponification treatment hydrolyzes AXT esters. 

### 2.2. Effect of AXT on IFN-γ Production in Splenocytes of H. pylori-Inoculated Mice

**Figure 2:**
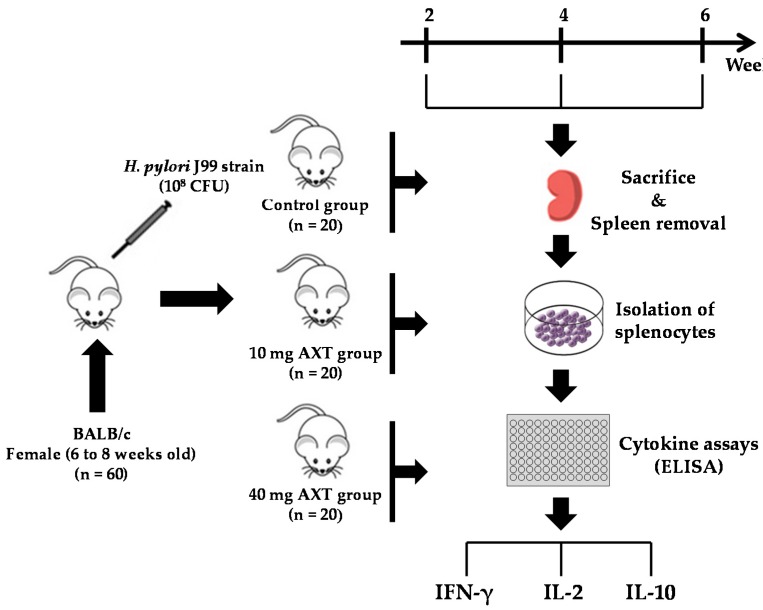
Experimental design to investigate the effects of AXT from shrimp cephalothorax on the cytokine release of splenocytes in *H. pylori*-infected mice.

**Figure 3:**
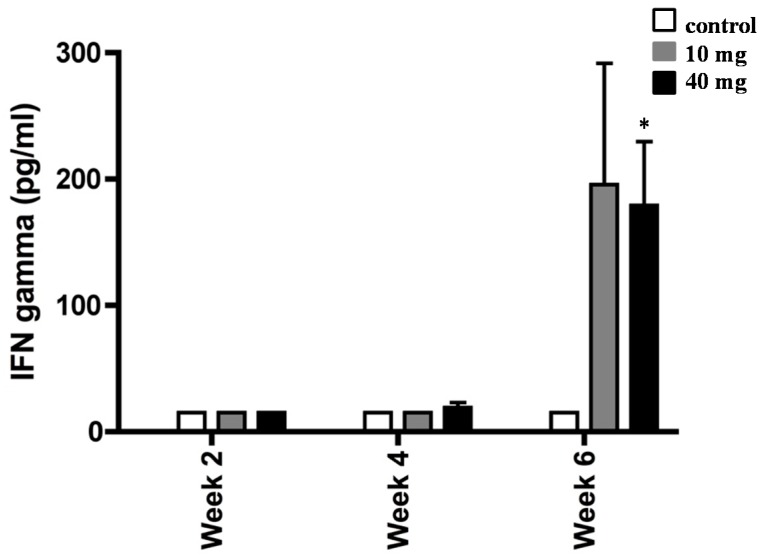
Interferon gamma (IFN-γ) production by splenocytes from *H. pylori*-infected mice. BALB/c mice were daily treated with 10 or 40 mg of AXT and sacrificed two, four, and six weeks post-inoculation. Splenocytes from infected mice were stimulated with sonicated *H. pylori* (50 µg mL^−1^), harvested after 36 h, and used for ELISA. Values are the mean ± SEM of duplicate ELISA determinations. Only values within a detection limit of 15–2000 pg/mL are represented. * *p* < 0.05 versus 10 mg and control groups.

The experimental design is shown in Figure 2. First, we determined the ability of splenocytes from *H. pylori*-inoculated and control mice to produce IFN-γ following supplementation with 10 and 40 mg of AXT (Figure 3).

Cells from *H. pylori*-infected mice did not produce IFN-γ until four weeks post initial consumption of AXT. Although the values were not significant, we observed a slight trend toward the upregulation of IFN-γ with the highest concentration of AXT after four weeks of treatment. However, following six weeks of treatment, splenocytes produced markedly elevated levels of IFN-γ (*p* < 0.05) in *H. pylori*-infected mice orally dosed with 40 mg of AXT.

### 2.3. AXT Influences IL-10 Production in Splenocytes of H. pylori-Inoculated Mice

**Figure 4:**
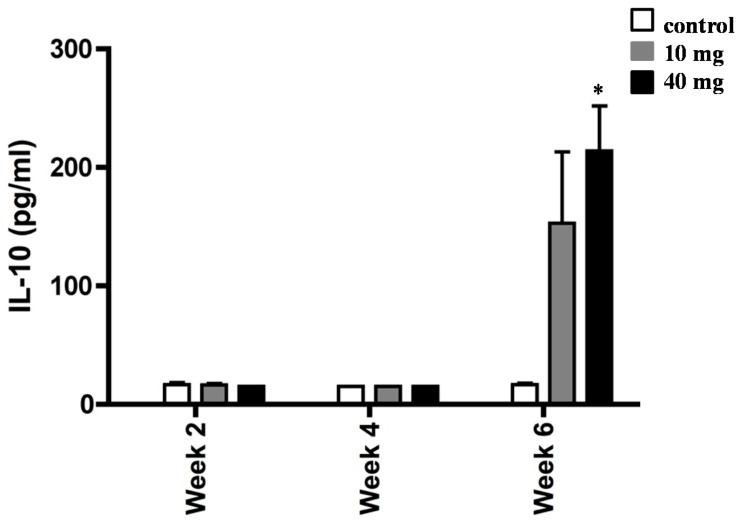
Induction of IL-10 release of splenocytes from *H*. *pylori*-infected BALB/c mice. Animals orally dosed with 10 and 40 mg of AXT were sacrificed two, four, and six weeks post-inoculation. Splenocytes from infected mice were stimulated with sonicated *H. pylori* (50 µg mL^−1^), harvested after 36 h, and used for ELISA. Data are presented as the mean ± SEM. Only values within a detection limit of 15–2000 pg/mL are represented. * *p* < 0.05 versus 10 mg and control groups.

To determine whether AXT was capable of modulating cytokine profile, we next measured the levels of IL-10 in splenocytes of *H. pylori*-inoculated mice (Figure 4). Animals treated with 10 mg and 40 of AXT produced a significant amount of IL-10 release after six weeks of treatment, as compared to the infected untreated mice. However, the IL-10 release from splenocytes treated with 10 mg of AXT was not statistically significant. In contrast, the IL-10 release of cells from the infected mice treated with 40 mg of AXT was significantly different (*p* < 0.05) from untreated animals and mice consuming the lower dose. 

### 2.4. Effect of AXT on IL-2 Release in Splenocytes of H. pylori-Inoculated Mice

**Figure 5:**
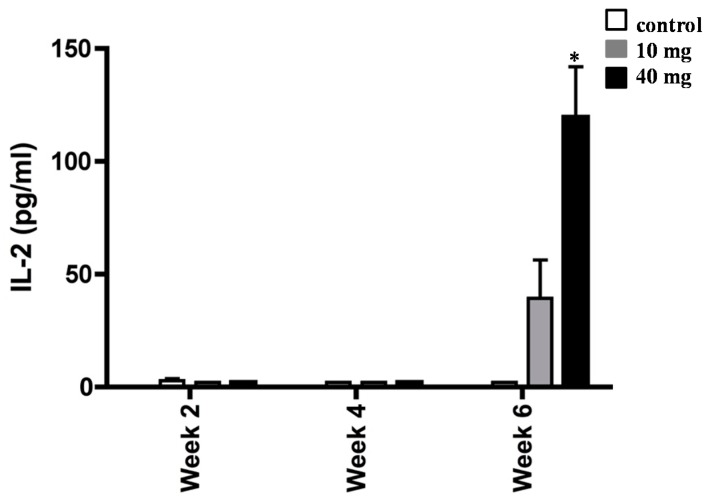
Increase of IL-2 synthesis of splenocytes from *H*. *pylori*-infected BALB/c mice. Animals daily dosed with 10 and 40 mg of AXT were sacrificed two, four, and six weeks post-inoculation. Splenocytes from infected mice were stimulated with sonicated *H. pylori* (50 µg mL^−1^), harvested after 36 h, and used for ELISA. Data are presented as the mean ± SEM. Only values within a detection limit of 15–2000 pg/mL are represented. * *p* < 0.05 versus 10 mg and control groups.

To extend the above findings that AXT played a key role in mediating *H. pylori*-associated inflammation, we next measured the release amount of IL-2 in splenocytes of *H. pylori*-inoculated mice supplemented with 10 and 40 mg of AXT (Figure 5). Although no statistical significance was observed, the synthesis of IL-2 was increased in splenocytes of *H. pylori*-inoculated mice treated with 10 mg of AXT. However, after six weeks, the level of IL-2 was significantly greater (*p* < 0.05) in animals treated with 40 mg of AXT than that of mice supplemented with 10 mg AXT or untreated animals. 

## 3. Discussion

Over the last few years, the consumption of dietary phytochemicals has been associated with health improvement and disease prevention [19,20,21,22,23]. Recently, AXT has been extensively studied due to its numerous interesting biological activities, including anti-microbial and anti-infection activities. Evidence from experimental and clinical models suggests that AXT possesses the ability to modulate the immune response, playing a crucial role against infectious diseases [24,25,26]. However, there is very little information on the immunomodulatory effects of AXT extracted from shrimps. Noteworthy, shrimp waste generated by shrimp processing industries is one of the cheapest raw materials to recover AXT. There are several infection diseases where immunostimulatory compounds are needed to inhibit the growth of bacteria and eradicate infection. The results of the current study suggest that AXT regulates the production of IFN-γ, IL-10, and IL-2 in splenocytes of *H. pylori*-infected BALB/c mice. In particular, it was shown that splenocytes from mice receiving the higher concentration of AXT produced more IFN-γ, IL-10, and IL-2 than those from control mice. These data are consistent with a previous study showing that *H. pylori* induces a predominant Th1 response and the release of IFN-γ, which was changed to a Th2 response and the release of IL-4 by AXT treatment [17]. Liu and Lee showed that a dietary cell extract from the microalgae *Chlorococcum* sp., which includes AXT, reduced the bacterial load and modulated cytokine production in *H. pylori*-infected BALB/c mice. These changes were associated with a shift of the T-lymphocyte response from a predominant Th1 response dominated by IFN-γ to a Th1/Th2 response with IFN-γ and IL-4 [27]. A study in *H. pylori*-positive patients with functional dyspepsia investigated gastric inflammatory markers and ILs (IL-4, IL-6, IL-8, IL-10, and IFN-γ) following treatment with AXT from *H. pluvialis*. There was a significant upregulation of CD4 and downregulation of CD8 in patients with *H. pylori* treated with AXT. However, cytokine levels in the infected tissues were not affected by AXT treatment [28]. We acknowledge that our study has a number of limitations, particularly the lack of analyses on gastric tissue samples. However, the results of the present study suggest that the administration of AXT from shrimp cephalothorax may modulate the cytokine profile during *H. pylori* infection. Human studies should be performed to determine whether AXT from the same source influences the expression of inflammatory mediators in *H. pylori*-infected tissues.

## 4. Materials and Methods

### 4.1. Mice and Housing 

*H. Pylori*-free BALB/c female mice, six- to eight-week-old, were purchased from Taconic (Germantown, NY, USA). The animals were housed in individually ventilated cages (IVC) (Techniplast, Buguggiate, Italy) to prevent airborne contamination. IVC feature a supply of high-efficiency particulate air (HEPA) and have been shown to be highly efficient as a barrier against bacteria [29]. The mice were randomly distributed with five animals in each cage and were kept in a room with a light:dark cycle of 12:12 h and with restricted staff access. During sampling and treatment, the appropriate cages were removed and an empty cage was replaced to maintain a constant airflow and pressure. Cages were only opened in a Laminar Air Flow (LAF) bench (Scanbur, Karlslunde, Denmark) and animals were always handled in here. Autoclaved chow, water, and bedding were provided to mice to reduce opportunistic infections. The mice were acclimatized two weeks before the start of the experiment. All animal experiments were approved by the National Board for Laboratory Animals (approval number: J. nr. 561-1163) and were conducted in accordance with applicable laws and regulations.

### 4.2. Helicobacter pylori Strain 

An *H. pylori* low passage J99 strain was kindly provided by Thomas Borén of Umeå University and was cultured on Columbia chocolate agar plates enriched with 7% defibrinated horse blood (SSI, Copenhagen, Denmark) under microaerobic conditions (10% CO_2_, 5% O_2_, and 85% N_2_) at 37 °C. After 48 h of growth, the cells were harvested in Brucella broth with 25% glycerol. The *H. pylori* strain J99, which has been fully sequenced, was isolated in the USA in 1994 from a patient with a duodenal ulcer that refused antimicrobial treatment [30].

### 4.3. Infection of Mice

Sixty mice were inoculated with 10^8^ colony forming units (CFU) of the *H. pylori* J99 strain three times at two-day intervals. A positive control group was comprised by mice which were infected with *H. pylori*, but neither treated nor handled in any way. All mice were daily attended twice by a veterinarian to ensure the animals’ welfare during the experiment. 

### 4.4. Preparation of Astaxanthin in the Lipid Extract 

AXT extracted from shrimp cephalothoraxes in a fish oil was used for this study. The extraction of AXT was performed according to established procedures, with slight modifications [31,32]. Briefly, more than 10 kilograms of frozen shrimp were collected from a local market. Shrimp cephalothoraxes were homogenized and aliquots of the homogenate were mixed with ethyl acetate and stirred for 30 min at room temperature in darkness; after extraction, the samples were filtered through Whatman No. 1 filter paper and all the aliquots were mixed together. Mono- and diesters of AXT were quantified and then the lipid extract was subjected to saponification for the complete hydrolysis of AXT esters. A portion of saponified sample was dried and re-dissolved in a known volume of acetone and subjected to purification of AXT on silica gel column.

### 4.5. Detection of Astaxanthin HPLC

The HPLC equipment used was a Shimadzu 20 series HPLC system equipped with a diode array detector. A 150 × 4.6 mm i.d. 5 μm C18 analytical column (Phenomenex) was used at 30 °C. A gradient mobile phase consisting of dichloromethane/methanol/acetonitrile/water (5.0:85.0:5.5:4.5, *v*/*v*, A) and dichloromethane/methanol/acetonitrile/water (25.0:28.0:42.5:4.5, *v*/*v*, B) was used for AXT and AXT esters analysis: 0 min, 100% A, 0% B; 8 min, 100% A, 0% B; 20 min, 0% A, 100% B; 24 min, 0% A, 100% B; 25 min, 100% A, 0% B; 32 min, 100% A, 0% B. The flow rate was 1 mL/min and the injection volume was 10 μL. The peak area of AXT was measured at a wavelength of 474 nm.

### 4.6. Treatment with Astaxanthin

The mice were divided into groups and received a daily oral dose of 10 or 40 mg of AXT adjusted to a final volume of 200 µL in the same fish oil used to extract AXT. The doses were selected on the basis of previous studies with AXT in an experimental model for *H. pylori* infection [16,17]. All mice were sacrificed at two, four, and six weeks after the initiation of treatment and tissue samples were removed. 

### 4.7. Isolation of Splenocytes

Splenocytes were washed out of the spleen with RPMI 1640 medium (Sigma, St. Louis, MO, USA) using a cell strainer and the plunger from a syringe. Cells were re-suspended in phosphate-buffered saline PBS (pH 7.2) and red blood cells were lysed with 0.17 M ammonium chloride lysing agent. Then, the splenocytes were re-suspended to a concentration of 4 × 10^7^ mL^−1^ in RPMI 1640 supplemented with 10% fecal calf serum, L-glutamine, penicillin, and streptomycin (Sigma, St. Louis, MO, USA).

### 4.8. Cytokine Release Assay

Splenocytes (4 × 10^5^ mL^−1^) were added to each well in a 96-well cell culture plate (Thermo Fisher Scientific, Copenhagen, Denmark) and stimulated with 50 µg mL^−1^ *H. pylori* sonicate and incubated with 5% CO_2_ for 36 h at 37 °C. Culture supernatants were harvested after 36 hours and stored at −20 °C until use. The concentrations of IFN-γ, IL-2, and IL-10 were measured by enzyme linked immunosorbent assay (ELISA) according to the instruction of the manufacturer (DuoSeT, Genzyme, Cambridge, MA, USA).

### 4.9. Statistical Analysis

Statistical analyses were performed using GraphPad Prism Version 4 (GraphPad Software, San Diego, CA, USA). Results are expressed as the mean ± SEM. Data were compared by the Student’s or paired *t*-test and are considered significant at values of *p* < 0.05.
